# Antioxidants as a Potential Target against Inflammation and Oxidative Stress in Attention-Deficit/Hyperactivity Disorder

**DOI:** 10.3390/antiox9020176

**Published:** 2020-02-21

**Authors:** Lourdes Alvarez-Arellano, Nadia González-García, Marcela Salazar-García, Juan Carlos Corona

**Affiliations:** 1 CONACYT-Hospital Infantil de México Federico Gómez, Mexico City 06720, Mexico; lourdes.alvareza@gmail.com; 2 Laboratory of Neurosciences, Hospital Infantil de México Federico Gómez, Mexico City 06720, Mexico; nadiag.him@gmail.com; 3 Laboratorio de Investigación en Biología del Desarrollo y Teratogénesis Experimental, Hospital Infantil de México Federico Gómez, Mexico City 06720, Mexico; msalazar.investigacion@gmail.com

**Keywords:** antioxidants, ADHD, inflammation, oxidative stress, Nrf2

## Abstract

Psychostimulants and non-psychostimulants are the medications prescribed for the treatment of attention-deficit/hyperactivity disorder (ADHD). However, several adverse results have been linked with an increased risk of substance use and side effects. The pathophysiology of ADHD is not completely known, although it has been associated with an increase in inflammation and oxidative stress. This review presents an overview of findings following antioxidant treatment for ADHD and describes the potential amelioration of inflammation and oxidative stress using antioxidants that might have a future as multi-target adjuvant therapy in ADHD. The use of antioxidants against inflammation and oxidative conditions is an emerging field in the management of several neurodegenerative and neuropsychiatric disorders. Thus, antioxidants could be promising as an adjuvant ADHD therapy.

## 1. Introduction

### 1.1. Attention-Deficit/Hyperactivity Disorder

Attention-deficit/hyperactivity disorder (ADHD) is the most common neurobehavioural disorder and is a chronic, often lifelong, condition [1,2]. The pooled worldwide prevalence of ADHD is 7.2% for children and adolescents and 3–5% for adults [3,4,5]. Boys are more than twice as likely as girls to receive a diagnosis of ADHD [6]. Approximately 50% of individuals diagnosed in childhood and adolescence persist with symptoms into adult life [1,7]. Comorbidity in ADHD is very common at roughly 70%, the main disorders being emotional or behavioural conditions, such as anxiety, oppositional defiant, depression and substance use disorders, and developmental conditions, such as learning and language disorders, autism spectrum disorders (ASD), and physical conditions (tics and sleep apnoea) [8,9,10]. The diagnosis of ADHD is based on the fifth edition of the Diagnostic and Statistical Manual of Mental Disorders (DSM-5) and the criteria include: inattention and/or hyperactive and impulsive symptoms for the last six months or more, onset before the age of 12 years old, and symptoms causing at least moderate psychological, social, and/or educational or occupational impairments based on interview and/or direct observation in multiple settings [6].

### 1.2. Pathophysiology

The pathophysiological mechanisms of ADHD are still not understood. However, biochemical, psychological, and environmental factors have generally been accepted as causes of the disorder. Several studies have suggested deregulation in catecholaminergic neurotransmission to be the cause [11,12]. Moreover, increasing evidence indicates a critical role for neuroinflammation [13,14]. Also, the involvement of oxidative stress is highlighted as a pathophysiological cause of ADHD [15,16].

### 1.3. Pharmacological Treatment

The management of ADHD comprises multimodal treatments that include psychosocial and educational interventions [6]. Nevertheless, pharmacological treatment is the first choice of therapy to improve symptoms, using psychostimulants such as methylphenidate (MPH) and amphetamines, which inhibit the reuptake of dopamine and norepinephrine, thus increasing catecholaminergic activity in the prefrontal cortex, striatum, and hippocampus, improving symptoms [6,17]. The second choice of therapy is with non-psychostimulants such as atomoxetine (ATX), which is a selective norepinephrine reuptake inhibitor, guanfacine, and clonidine, which are selective α-2 adrenergic receptor agonists [18,19].

However, psychostimulants have been associated with side effects such as appetite loss, headache, stomach pain, agitation, sleep disturbance, anxiety, and insomnia [2,20]. Moreover, these medications have a persistent effect on decreasing growth velocity and hallucinations or other psychotic symptoms [21]. Furthermore, non-psychostimulants are associated with changes in cardiovascular parameters, cardiovascular events, somnolence, gastrointestinal tract symptoms, nausea, diarrhoea, vomiting, decreased appetite, fatigue, and dizziness [2,22].

**Figure 1:**
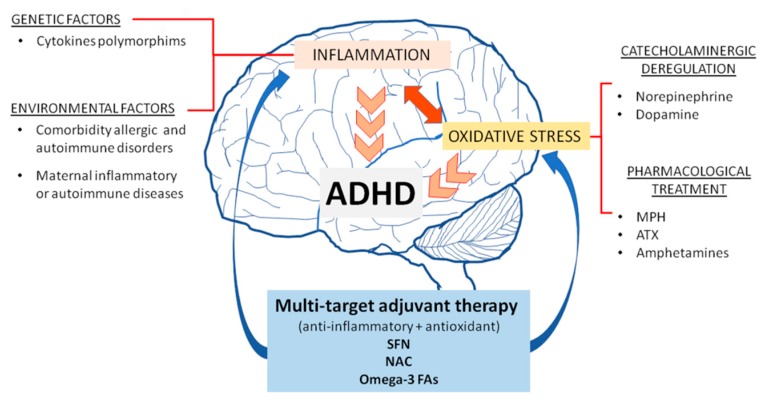
Role of inflammation and oxidative stress in the pathophysiology of ADHD and potential adjuvant therapy. Environmental and genetic factors, catecholaminergic dysregulation and pharmacological treatment can establish a vicious circle, producing inflammation and oxidative stress, therefore contributing to increase the symptoms. SFN, sulforaphane; NAC, N-Acetylcysteine; omega-3 FAs, omega-3 fatty acids; MPH, methylphenidate; ATX, atomoxetine.

Neuroinflammation and oxidative stress play a role in the pathophysiology of ADHD due to genetic and environmental factors, catecholaminergic dysregulation, and medications used for treatment, all factors which could produce inflammation and oxidative stress, which increases the symptoms and, as a consequence, leads to establishing a vicious circle (Figure 1). Hence, antioxidants against inflammation and oxidative stress have been used to manage other disorders such as Alzheimer’s disease, Parkinson’s disease, Huntington’s disease, autism, schizophrenia, and depression [23,24,25,26,27,28]. Accordingly, antioxidant modulators could be helpful as a multi-target adjuvant therapy in ADHD.

## 2. Inflammation and the Relationship with ADHD

Increasing evidence supports the role of neuroinflammation in the pathophysiology of ADHD [14]. Inflammation in the brain is characterized by the activation of glial cells (oligodendrocytes, astrocytes, microglia and ependymal cells) and the production of cytokines, chemokines, prostaglandins, nitric oxide (NO), reactive oxygen species (ROS), and immune cell infiltration, including monocytes/macrophages, neutrophils, dendritic cells, T cells, and B cells. Glial cells, principally microglia (brain-resident immune cells), are responsible for the maintenance of homeostasis after brain injury [29,30].

Increased ADHD symptoms have been reported in patients with an uncontrolled inflammatory environment. Thus, elevated levels of interleukin-6 receptor (IL-6R), RANTES (regulated upon activation, normal t cell expressed and secreted) and tumour necrosis factor-α (TNF-α) in children with ADHD were associated with a major intensity of symptoms such as hyperactivity and inattention [31]. Moreover, serum levels of IL-6 and IL-10 were significantly higher in ADHD children compared with healthy controls. However, IL-6 levels did not correlate with the severity of ADHD symptoms [32,33]. In an animal model of ADHD, using spontaneously hypertensive rats (SHR), the serum levels of IL-1α, MCP-1, RANTES, and IP-10 were elevated in five-week-old compared to control rats. Interestingly, serum levels of IL-6 were similar in five-week-old animals of both strains, with elevated levels in 10-week-old SHR, which correlates with that found in children with ADHD [34]. The association between the dysregulation of the inflammatory response and the pathophysiology of ADHD is possible as a result of the role of inflammation in neurogenesis, differentiation, and neuronal function [30,35,36,37]. Furthermore, neuroinflammation can induce aggravating factors such as blood–brain barrier disruption, altered neurotransmitter metabolism, oxidative stress, and neurodegeneration [38].

The inflammatory mechanisms and the association of dysregulation in ADHD remain to be fully clarified but involve genetic and/or environmental factors. Several studies have reported an association between ADHD and the polymorphism of cytokines such as IL-2, IL-6 and TNF-α [39]. However, there are controversial results for interleukin-1 receptor antagonist (IL-1RA; also known as IL-1RN) gene variable number tandem repeat (VNTR) polymorphism: the four-repeat allele was associated with increased risk and the two-repeat allele with reduced risk for ADHD [40]. On the other hand, no association of this IL-1RN polymorphism with ADHD was found in a larger sample [41]. Comorbidity with allergic and autoimmune disorders such as atopic eczema, allergic rhinitis and asthma is another factor that could increase the risk for ADHD and future research may lead to a better understanding of the mechanisms underlying the observed comorbidity [38,42,43,44]. In a meta-analysis and a large-scale genome-wide association study (GWAS), associations between asthma and ADHD were found in both children and adults [45,46]. Also, patients with ADHD and asthma had similar brain region dysfunctions and higher levels of cytokines and IgE compared to healthy children, resulting in alterations to the regions in the brain associated with emotional and behavioural control [38,42,47]. The involvement of autoantibodies in ADHD has been suggested but this is an unknown causal association [33,48]. High antibody levels of anti-Purkinje cells (anti-Yo), anti-basal ganglia and anti-dopamine transporter were found in patients with ADHD [33,48,49,50]. Moreover, elevated anti-Yo antibody levels were correlated with IL-6 and IL-10 levels [33]. Further studies are needed to clarify the comorbidity or causal relationship between allergic and autoimmune disorders and ADHD. Maternal history of autoimmune disease has also been associated with an increased risk of ADHD. The inflammation caused during prenatal development by several maternal diseases, such as infections, asthma, diabetes, obesity, and autoimmune disease, was associated with ADHD in offspring [13,51].

## 3. Antioxidant Treatment Against Inflammation in ADHD

### 3.1. Sulforaphane Exerts Anti-Inflammatory Activity

Sulforaphane (SFN) is found in highest concentrations in vegetables such as cauliflower and broccoli sprouts, and it has been shown that SFN is an activator of nuclear factor erythroid 2-related factor 2 (Nrf2) [52]. Nrf2 is a transcription factor widely recognized as a master regulator of cellular redox homeostasis [53,54]. Regulation is carried out by binding Nrf2 to a specific DNA sequence known as the antioxidant response element (ARE) found in the promoter regions of genes that encode detoxification enzymes such as NADPH quinone oxidoreductase 1 (NQO1), haem oxygenase 1 (HO-1) and glutathione peroxidase 1 (GPx1), among others [55]. Nrf2 regulates enzymes responsible for GSH syntheses, such as the glutamate-cysteine catalytic subunit (GCLC) and glutamate-cysteine ligase modifier subunit (GCLM), and enzymes related to GSH utilization, such as glutathione S transferase (GST), glutathione peroxidase, and glutathione reductase [54]. Thus, SFN activates Nrf2 and stimulates transcription of genes involved in GSH synthesis.

**Figure 2:**
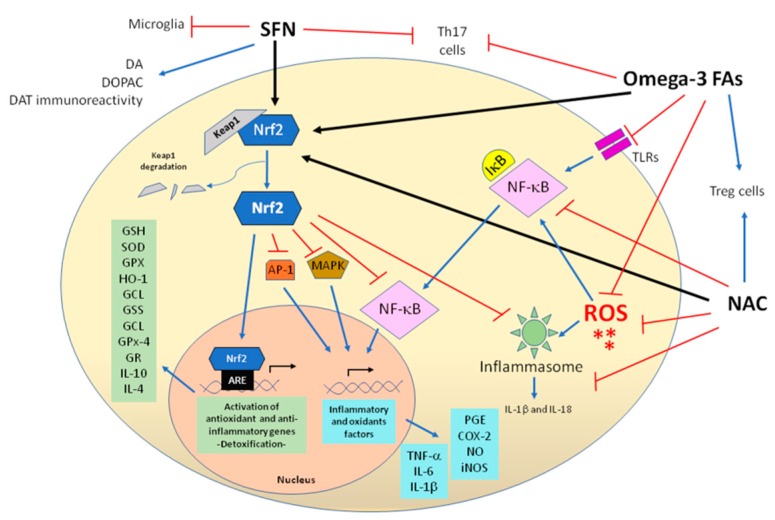
Overview of the mechanisms of SFN, NAC and Omega-3 FAs used as modulators against inflammation and oxidative stress. DA, dopamine; DOPAC, 3,4-dihydroxyphenylacetic acid; DAT, dopamine transporter; PGE, prostaglandins; ROS, reactive oxygen species; NO, nitric oxide; GCL, glutamate cystine ligase; GPx, glutathione peroxidase; GSH, glutathione; SOD, superoxide dismutase; GR, glutatione reductase; GSS, glutathione synthetase; glutathione peroxidase-4, GPx-4.

SFN has been considered as a therapeutic target in several inflammation-associated diseases, including neurodevelopmental disorders such as psychosis and autism spectrum disorder [56,57,58]. Action mechanisms of antioxidants used against inflammation are shown in Figure 2.

The molecular mechanism by which SFN exerts its anti-inflammatory function is by inducing Nrf2 pathway activation, which contributes to the anti-inflammatory process by regulating HO-1 gene expression [59,60,61]. Nrf2 leads to the inhibition of nuclear factor kappa B (NF-κB), activator protein-1 (AP-1) and mitogen-activated protein kinase (MAPK) classical inflammatory pathways, resulting in decreased expression of the inflammatory mediators (iNOS, COX-2, NO and prostaglandins) and pro-inflammatory cytokines (TNF-α, IL-6 and IL-1β). In addition, Nrf2/HO-1 activation increases anti-inflammatory cytokines (IL-10 and IL-4) [62,63,64,65]. Moreover, SFN has a prophylactic and a therapeutic effect by inhibiting both the inflammatory response and microglial activation [62]. 

Inflammasomes are multiprotein complexes necessary for the release of pro-inflammatory cytokines (IL-1α and IL-18). Interestingly, it has been reported that SFN inhibited the activation of NLRP1, NLRP3, and NLRC4 inflammasomes [66,67]. However, the precise mechanism by which it does so is controversial, inasmuch as the SFN-mediated inhibition of the inflammasomes was independent of caspase-1 activity, ROS modulation, and Nrf2 synthesis [66]. Contrary to these findings, other studies showed that ROS generation and Nrf2 were required for inflammasome activation [67,68]. SFN protected against neuroinflammation by preventing the increase of NF-κB activity, TNF-α, and depletion of the IL-10 level in the cortex and hippocampus of okadaic-acid-treated rats by the activation of the Nrf2/HO-1 pathway [69]. Recently, it was shown that treatment with SFN in ASD-induced improvement of social interaction and behavioural deficits may be due to the inhibition of STAT3 expression and suppression of Th17 cell response [27,70]. Although SFN supplementation in patients with ADHD has not been studied, this could constitute a promising approach against inflammation linked with ADHD. Nevertheless, more studies are needed to confirm safety, efficacy, therapeutic doses, effect in combination with conventional therapy and long-term side effects to be considered as adjuvant therapy in ADHD. The effects of antioxidants and the main outcomes are summarized in Table 1.

### 3.2. N-Acetylcysteine Decreases Inflammatory Response

N-Acetylcysteine (NAC), a precursor of L-cysteine and the antioxidant glutathione (GSH), is found in plants, especially the onion [96,97,98]. NAC has been used as an adjuvant therapy in many psychiatric disorders (e.g., Alzheimer´s disease, schizophrenia, autism, addiction, substance abuse, obsessive-compulsive and mood disorders [24,99,100,101,102,103,104]), with promising results and no relevant side effects after its administration against inflammation [97]. The use of NAC in a chronic unpredictable mild-stress animal model inhibited the levels of pro-inflammatory cytokines (IL-1β, IL-6, and TNF-α) in the hippocampus and prefrontal cortex and exhibited antidepressant-like effects [71]. However, in a recent study, NAC treatment did not show any effect on the serum levels of IL-6, IL-8, TNF-α, IL-10, or C-reactive protein in a clinical trial for bipolar depression, possibly due to the small sample size used in this study [72]. On the other hand, a study derived from an ADHD self-report scale symptom checklist revealed that NAC reduced the ADHD symptoms in patients with systemic lupus erythematosus and also inhibited the autoimmune inflammatory process via suppression of the mammalian target of rapamycin (mTOR) and increased regulatory T cells [73,74]. Moreover, NAC has been reported to block inflammasome activation as well as IL-18 and IL-1β production [75]. Recently, NAC protected against cisplatin-induced toxicity in rat brain by modulation of inflammation and oxidative stress [76]. Thus, more studies are required to support the efficacy of NAC as a possible adjuvant treatment for ADHD.

### 3.3. Omega-3 Fatty Acids Prevent Inflammation

Omega-3 fatty acids (omega-3 FAs) are polyunsaturated fatty acids, whose primary sources are in oily fish. They are components of neuronal membranes and have a main role in neurotransmission, neuronal development and function [105]. Two of the main omega-3 FAs are docosahexaenoic acid (DHA) and eicosapentaenoic acid (EPA) and it has been demonstrated that supplementation with omega-3 FAs has beneficial effects in several neurodegenerative and neuropsychiatric disorders such as Parkinson´s and Alzheimer´s diseases, depression, bipolar disorder, anxiety, and schizophrenia [106,107]. These omega-3 FAs are also known to have anti-inflammatory effects [108,109]. It has been demonstrated that DHA decreased the TLR-dependent inflammatory signalling pathway by inhibiting dimerization and the recruitment of receptors to lipid rafts, which resulted in the reduced production of pro-inflammatory cytokines [77,110]. DHA also reduces T-cell activation, proliferation and promoted polarization into regulatory T cells (Treg; CD4^+^/CD25^+^/FoxP3^+^) and interferes with the polarization of Th17 cells [78,79,80]. In a double-blind study, supplementation with omega-3 FAs for eight weeks was shown to decrease the plasma IL-6 level and any hyperactivity symptoms in children with ADHD [81]. However, a similar eight-week study found no effect on ADHD, but this could be due to the dose of omega-3 FAs [111]. Thus, further studies are needed to confirm the therapeutic dose, safety, and effectiveness of omega-3 FAs as a possible therapy against inflammation in ADHD.

## 4. Oxidative Stress and the Relationship with ADHD

There is increasing evidence for the involvement of oxidative stress in the pathophysiology of ADHD [15,16], but some studies have shown low levels of malondialdehyde (MDA) and the DNA damage indicator 8-hydroxy-2′-deoxyguanosine (8-OHdG) in ADHD children [112,113]. MDA is the degradation product of the main chain reactions that lead to the oxidation of polyunsaturated fatty acids, and therefore serves as an oxidative stress marker. Recently, it was reported that the total antioxidant capacity, catalase and GSH were significantly lower but that MDA was not significantly different in children with ADHD [114]. However, high levels of MDA have been observed in both adults [115,116] and children with ADHD [117], and increased plasma MDA and urinary 8-OHdG levels were found in children with ADHD compared to healthy children [118]. Another study has shown low total antioxidant levels in children with ADHD [119]. Furthermore, the SHR model showed an increase in ROS production in the hippocampus, cortex, and striatum [120]. It was shown that paraoxonase-1 (PON1) and arylesterase activity were decreased (the arylesterase activity linked to PON1 is known to protect lipoproteins from oxidation) and there was also a decrease in the total antioxidant status. Moreover, the total oxidant status and oxidative stress index were increased in children with ADHD, suggesting that there is significantly increased oxidative stress in ADHD [121]. It seems that nitrosative stress also has a role in ADHD because of increased oxidative and nitrosative stress and impaired oxidant–antioxidant balance has been demonstrated in children with ADHD [122]. The NO levels were significantly higher in children, adolescents and adults [117,123] and also significant increases in NO synthase activity were observed in children and adolescents with ADHD [124]. Although there are inconsistent results regarding the relationship of oxidative stress and ADHD, in a meta-analysis it was confirmed that ADHD is associated with increased oxidative stress in ADHD patients [16]. Taken together, the data suggest that both oxidative and nitrosative stress in ADHD have the potential to contribute to this condition [15]. 

On the other hand, it has been shown that dopamine and norepinephrine can easily undergo auto-oxidation, forming ROS [125,126], which could lead to cell damage and damage to DNA [127,128]. Also, it has been shown that ATX treatment increases extracellular norepinephrine and dopamine levels [19,129], which would produce an increase in oxidative stress and, as a consequence, cell damage and mitochondrial dysfunction [130]. The brain is particularly susceptible to oxidative stress because of its high lipid content and the high demand for energy consumption [131]. Neurons use mitochondria as the main producers of ATP. Mitochondria regulate ion homeostasis and the redox state, and they are also both producers of ROS and targets of ROS-induced damage, with such effects leading to the collapse of bioenergetic function and the initiation of cell death [132]. For this reason, oxidative stress could also be involved or interrelated with the catecholaminergic pathway in ADHD. However, the exact relationship between both processes and ADHD remains unclear. 

Regarding ADHD medication, several reports have demonstrated that MPH has an impact on the generation of oxidative damage. Increases in DNA damage have been found mainly in the striatum of young and adult rats after MPH treatment [133]. In specific regions of the brain of young rats, chronic treatment with MPH increased oxidative damage, as assessed by the thiobarbituric acid reactive species and protein carbonyl assays, and this effect was dependent on the dose [134]. Furthermore, acute or chronic treatment with MPH altered the activity of catalase and superoxide dismutase (SOD) enzymes in the brain of young rats [135]. One study showed that acute and chronic treatment with MPH in the SHR model increased oxidative stress [136]. Finally, the acute administration of high doses of MPH can cause oxidative and inflammatory changes in brain cells and induce neurodegeneration in the hippocampus and cerebral cortex of adult rats [137].

Hence, the growing research in looking for alternative therapies for ADHD has focused on the neuroprotective effects of natural products as antioxidants because they may be high-efficiency alternative treatments with fewer side effects [2].

## 5. Antioxidant Treatment Against Oxidative Stress in ADHD

As a consequence of oxidative stress, cells have the capacity to increase their antioxidant defences through the activation of Nrf2. Thus, Nrf2 pathway activation takes place to act against the accumulation of ROS. Consequently, Nrf2 activators have been proposed as antioxidant targets in neurodegenerative and neuropsychiatric disorders (Alzheimer’s disease, Parkinson’s disease, Huntington’s disease, autism, schizophrenia, and depression) to counteract the increase in oxidative stress [138,139,140]. The action mechanisms of the antioxidants used against oxidative stress are shown in Figure 2.

### 5.1. Sulforaphane Exerts Antioxidant Activity

In mice, the repeated administration of amphetamines induced decreases of dopamine and 3,4-dihydroxyphenylacetic acid DOPAC levels, as well as dopamine transporter immunoreactivity in the striatum, and such effects were significantly attenuated by the treatment with SFN [82]. Activation of the cellular antioxidant machinery (HO-1, glutamate-cysteine ligase catalytic subunit and Nrf2) resulted in SFN-mediated protection against memory impairment in rats treated with okadaic acid [69]. Moreover, in a mouse model of autism, SFN improved the autism-like symptoms and upregulated antioxidant defences such as SOD, glutathione reductase, and GPx [70]. The combination of NAC and SFN significantly reduced oxidative stress, delayed the onset of epilepsy, blocked disease progression, and reduced the frequency of spontaneous seizures in animals [83]. Also, in a clinical pilot study, treatment with SFN increased the antioxidant GSH, suggesting a need to explore possible correlations between GSH and clinical/neuropsychological measures and any positive influence that the treatment of SFN could have on neuropsychiatric disorders [26]. Even though the antioxidative effect of SFN supplementation in ADHD has not been studied, this could constitute a promising approach for oxidative imbalances linked with ADHD. Nevertheless, more research is needed to confirm the efficacy, therapeutic doses, and effect in combination with conventional therapy to be considered as adjuvant therapy in ADHD. The effects of antioxidants and the main outcomes are summarized in Table 1.

### 5.2. N-Acetylcysteine Exerts Antioxidant Activity

The NAC molecule scavenges ROS and there has been growing evidence of its role in attenuating psychiatric and neurological disorders and associated pathophysiological processes such as oxidative stress, mitochondrial dysfunction and glutamate and dopamine dysregulation [96,97,141]. In rats, amphetamine produces oxidative stress (by increasing hydroxyl radical formation and MDA) and dopaminergic neurotoxicity, but treatment with NAC protected against amphetamine-induced damage [84]. The treatment of paediatric Tourette´s syndrome with NAC in a randomized, double-blind placebo-controlled trial did not show a significant difference between NAC and placebo for reducing tic severity or any secondary outcomes such as depression, anxiety and ADHD [85]. In a case-study, a 17-year-old girl was successfully treated with NAC, reducing the frequency of self-cutting and the symptoms of ADHD and depression [86]. Finally, in a mouse model of postoperative cognitive dysfunction, NAC reduced oxidative stress and inflammation in the hippocampus and improved cognitive function by activation of the Nrf2/HO-1 pathway [87]. Thus, it seems that part of the protection produced by NAC is via the activation of antioxidant pathways that involve Nrf2. However, more research is required to support the efficacy of NAC as a possible treatment for ADHD.

### 5.3. Omega-3 Fatty Acids Exert Antioxidant Activity

The omega-3 FAs supplementation can be an enhancer factor in the antioxidant defence against ROS [142] and has been demonstrated to be effective against oxidative stress in the treatment of ADHD. A pilot study demonstrated that supplementation with alpha-linolenic acid, an omega-3 FAs, improved ADHD symptoms [91]. The findings of a small pilot study demonstrated that supplementation with high doses of EPA and DHA improved the behaviour of children with ADHD [92]. In a double-blind study, eight weeks of EPA and DHA supplementation decreased oxidative stress in children with ADHD [81]. Supplements with high EPA, DHA or omega-6 FAs as a control demonstrated no significant differences among the treatments. However, in one subgroup of children there was improvement in spelling, reading and attention and a decrease in hyperactivity [88]. On the other hand, in one study, EPA and DHA supplementation in children with ADHD demonstrated no significant improvements in outcome [89]. Furthermore, no beneficial results were observed in a randomized placebo-controlled clinical trial with EPA and DHA supplementation in children with ADHD [90]. There were increased EPA and DHA concentrations in erythrocyte membranes and improved working memory function on supplementation with a mix of omega-3 FAs [93]. The effects of dietary omega-3 FAs supplementation on ADHD showed a reduction in ADHD symptoms [94]. Finally, omega-3 FAs improved the antioxidant defence (by increasing glutamate-cysteine ligase, Nrf2, glutathione synthetase and glutathione peroxidase-4 proteins) in astrocytes treated with hydrogen peroxide, and Nrf2 activation was dependent on the proportion of DHA to EPA incorporated into the membrane phospholipids [95]. Thus, omega-3 FAs could be improving the antioxidant defences at least in part through the activation of the Nrf2 pathway.

## 6. Conclusions

The association of ADHD with an increase in inflammation and oxidative stress could play a role in the pathophysiological process. Nowadays, ADHD has no therapeutic option able to counteract the progression of the disorder, and therapy with MPH and amphetamines might be increasing oxidative stress. Thus, all evidence points towards inflammation and oxidative stress as factors which are influencing ADHD, whereas antioxidants may perhaps be able to ameliorate ADHD progression due to their anti-inflammatory and antioxidant properties. Consequently, some of the antioxidants discussed in this review might establish a new therapeutic approach for the treatment of ADHD. While the use of natural antioxidants for diverse disorders has been considered as a safe and healthier approach for patients, they are still far from being standard treatments, due to the lack of controlled clinical studies that may well corroborate both their high efficacy and safety. Accordingly, better designed and more rigorous research and clinical trials are required before they can be established as a therapeutic alternative and further studies would also be necessary to corroborate the use of these antioxidants administered as a co-treatment with the current medications. Nevertheless, antioxidants could be considered as a multi-target adjuvant therapy for ADHD.

## Figures and Tables

**Table 1:** Summary of the findings on the potential beneficial effects of antioxidants against inflammation and oxidative stress.

**Against Inflammation**	**Type of Study**	**Outcome**
SFN	Mouse model of atopic dermatitis [61]	Reduced inflammation, suppressed JAK1/STAT3 signaling and activated Nrf2/HO-1 pathway
SFN	Microglial cells [62]	Reduced inflammatory mediators (iNOS, COX-2, NO, and PGE2) and proinflammatory cytokines (TNF-α, IL-6, and IL-1β), increased anti-inflammatory cytokines (IL-10 and IL-4) and increased the expression of Nrf2 and HO-1.
SFN	Mouse model of acute lung injury and Macrophages [63,65]	Decreased lactate dehydrogenase, IL-6, TNF-α, NF-kB, PGE2 production, COX-2, MMP-9 and iNOS protein expression
SFN	Mouse model of peritonitis [66]	Inhibited inflammasome activation and IL-1β secretion and inhibited cell recruitment to peritoneum.
SFN	Mouse macrophages [67]	Blocked activation of NLRP3 and NLRC4 inflammasomes and IL-1β secretion
SFN	Rat [69]	Inhibited NF-kB activity and TNF-α secretion and prevent decreased IL-10
SFN	Mouse model of autism and Autism patients [27,70]	Reduced Th17 response and expression of NF-kB and iNOS Randomized double-blind study; decreased symptoms
NAC	Mild-stress rat model [71]	Inhibited pro-inflammatory cytokines (IL-1β, IL-6 and TNF-α)
NAC	Bipolar depression patients [72]	Randomized placebo-controlled trial; no effects on the biological parameters evaluated
NAC	Systemic lupus erythematosus patients [73,74]	Randomized double-blind placebo-controlled trial and randomized controlled trial; reduced the ADHD symptoms and also inhibited the autoimmune inflammatory process by suppression of the mammalian target of rapamycin (mTOR) and increased regulatory T cells
NAC	Human retinal pigment epithelial cell line [75]	Decreased IL-18, IL-1β mRNA, ROS and blocked inflammasome activation
NAC	Rat [76]	Improved brain oxidant/antioxidant status and reversed the overproduction of pro-inflammatory cytokines in brain and serum
Omega-3 FAs	Macrophage and mouse dendritic cell lines [77,78]	Inhibited dimerization and recruitment of TLR2 and TLR4 recruitment to lipid rafts and reduced T-cell proliferation and increased the proportion of T cells expressing FoxP3
Omega-3 FAs	Mouse [79,80]	Regulated CD4^+^ T-cell function and reduced Th17cell polarazation
Omega-3 FAs	Children with ADHD [81]	Double-blind study; decreased plasma inflammatory mediators
**Against Oxidative Stress**	**Type of Study**	**Outcome**
SFN	Mouse [82]	Increased dopamine, DOPAC and dopamine transporter immunoreactivity in the striatum
SFN	Rat [69]	Activation of HO-1, glutamate-cysteine ligase catalytic subunit and Nrf2 and protected against memory impairment
SFN	Mouse model of autism [70]	Improved the autism-like symptoms and upregulated SOD, glutathione reductase and GPx
SFN and NAC	Rat with epilepsy [83]	Reduced oxidative stress, delayed the onset of epilepsy, blocked disease progression and reduced the frequency of spontaneous seizures
SFN	Healthy subjects [26]	Clinical pilot study; increased GSH
NAC	Rats [84]	Protected against amphetamine-induced damage
NAC	Paediatric Tourette’s syndrome [85]	Randomized double-blind placebo-controlled trial; did not show a significant difference with placebo
NAC	A girl with ADHD [86]	A case-study; reduced the frequency of self-cutting and reduced the symptoms and depression
NAC	Mouse model of postoperative cognitive dysfunction [87]	Reduced oxidative stress and inflammation in the hippocampus and improved cognitive function by activation of the Nrf2/HO-1 pathway
Omega-3 FAs	Children with ADHD [81]	Double-blind study; decreased oxidative stress
Omega-3 FAs	Children with ADHD [88]	Randomized controlled trial; no significant differences among the treatments. One subgroup improved spelling, reading and attention and decreased hyperactivity
Omega-3 FAs	Children with ADHD [89,90]	Randomized pilot study and placebo-controlled trial; no significant improvement
Omega-3 FAs	Children with ADHD [91,92,93,94]	Pilot studies and randomized placebo-controlled trials; improved working memory function and improved symptoms and behaviour
Omega-3 FAs	Rat astrocytes [95]	Increased glutamate-cysteine ligase, Nrf2, glutathione synthetase and glutathione peroxidase-4 proteins
